# Advances in artificial intelligence for the early detection of cervical cancer in adult women: a scoping review

**DOI:** 10.61622/rbgo/2025rbgo76

**Published:** 2025-11-18

**Authors:** Laura Vanesa Henao Ramírez, Hanna Eljach Forero, María Paula Novoa Grosso, Erwin Hernando Hernández Rincón

**Affiliations:** 1 Universidad de La Sabana School of Medicine Chía Colombia School of Medicine, Universidad de La Sabana, Chía, Colombia.; 2 Universidad de La Sabana School of Medicine Department of Family Medicine and Public Health Chia Colombia Department of Family Medicine and Public Health, School of Medicine, Universidad de La Sabana, Chia, Colombia.

**Keywords:** Adult women, Cervical cancer screening, Cervical cancer, Cervicovaginal cytology, Papanicolaou test, HPV-based screening, Artificial intelligence

## Abstract

**Objective:**

To synthesize current scientific evidence on the benefits and potential contributions of integrating AI-based technologies with traditional diagnostic methods used for the detection and early diagnosis of cervical cancer in adult women.

**Methods:**

An exhaustive search was conducted in academic databases (PubMed, Scopus and BIREME) using specific search terms and Boolean operators in December 2024. Independently conducted by three researchers across all databases, the selection process included articles involving adult women with either suspected or confirmed cervical cancer, in which the application of artificial intelligence (AI) technologies was examined across various techniques used for early diagnosis of the disease, such as cytology, colposcopy, and radiological imaging, among others. Only articles published between 1999 and 2022 were included. The included articles were reviewed in full text by all authors, and relevant data were extracted and organized into a chart comprising the following items: author and year of publication, title, study design, type of AI technology used, and a summary of the content.

**Results:**

AI, particularly through Machine Learning (ML) algorithms, demonstrated significant improvements in the accuracy, sensitivity, and efficiency of cervical lesion classification when combined with conventional diagnostic techniques like cervical cytology, colposcopy, and biopsy. This combined approach outperformed traditional methods used in isolation.

**Conclusion:**

The integration of AI with standard cervical cancer screening and diagnostic methods offers substantial benefits, including faster detection times, reduced workload for pathologists, and improved patient outcomes by facilitating earlier treatment initiation and reducing diagnostic variability. Considering the available literature, the use of AI may offer potential benefits; however, further studies are required.

## Introduction

Cervical cancer is a type of malignant neoplasm that originates in the uterus at the cervix, and at the squamocolumnar junction, affecting the external squamous epithelial cells and/or the internal glandular epithelium.^(1)^ It is the fourth most common cancer in women worldwide, with an incidence of 13.3 cases per 100,000 women per year and a mortality rate of 7.2 deaths per 100,000 women per year documented in 2022.^(2)^ Human Papillomavirus (HPV) is globally recognized as the primary etiological agent of this condition, with serotypes 16, 18, and 33 having the highest oncogenic potential and currently being prevalent in women who have not been vaccinated against this microorganism. This infection is asymptomatic, which has led to the implementation of screening strategies, the most widely used being cervicovaginal cytology, which allows for a morphological study of epithelial cells, and the HPV DNA detection test, which is currently considered the gold standard for early identification of cervical cancer risk due to its high sensitivity and predictive value. Finally, the diagnosis is confirmed through biopsy, which allows for the evaluation of histological changes characteristic of malignancy.^(3,4)^ However, a delay in the diagnosis of cervical cancer has been documented in 4.3% to 89.1% of patients, attributable to the limitations of human evaluators in detecting early neoplastic changes during this process.^(5,6)^

This limitation, observed across various medical pathologies, has driven the development of technologies based on machine learning (ML) algorithms, capable of processing large volumes of clinical data, leading to generative and integrative models. These tools have proven to improve performance and diagnostic accuracy by early identification of changes associated with multiple diseases, including cancer. By assisting healthcare professionals in decision-making, these technologies optimize both the accuracy and speed of diagnosis (Chart 1).^(7)^

**Chart 1:** Terminology in Artificial Intelligence (AI)

Terminology in Artificial Intelligence (AI)^(8,9)^	
Machine learning (ML)	A subset of AI focused on developing models and algorithms that enable machines to automatically improve and learn patterns without explicit human intervention.
Deep learning (DL)	A subdiscipline of Machine Learning with a focus on automated learning that uses artificial neural networks with multiple hidden layers to perform complex data processing tasks such as image recognition, natural language processing, and speech recognition.
Neural Networks	These are networks of processing units called neurons, which are organized in layers and connected by weighted connections that allow the network to learn using input data. Their name is derived from the inspiration taken from the structure of the human brain, and they were designed to perform tasks of machine learning.
Convolutional Neural Networks (CNNs)	A specialized type of neural network primarily used for image processing and computer vision. They allow the extraction of meaningful features from images.
Particle Swarm Optimization (PSO)	An algorithm in which a group of particles (potential solutions) explore different areas to find the best option. Each particle adjusts its position based on what it learns from its own experience and other particles, helping to find solutions quickly and efficiently. It is inspired by the way groups of animals, such as birds or fish, move.
Automated Visual Assessment (AVA)	A field focused on enabling computers to interpret images and videos. To understand visual content, it utilizes techniques from image processing, machine learning, and computer vision.

In the context of cervical cancer, recent advancements in artificial intelligence (AI) offer considerable prospects for automated, objective, and impartial detection of cervical cancer and precancerous conditions.^(6)^ A study published in 2024 analyzed 32 studies conducted between 2009 and 2022 that integrated images, such as digital colposcopy and cervigraphy, with various ML and deep learning algorithms.

Among these, Support Vector Machines (SVM), Convolutional Neural Networks (CNN), Residual Networks (ResNet), and Visual Geometry Group (VGG) models stood out, achieving the best diagnostic approximation performances with accuracies exceeding 97%.^(10)^ Although the ability of these technologies to improve performance in screening and early diagnosis has been demonstrated, the current literature does not provide a clear synthesis regarding the utility of this technology in early diagnostic confirmation, specifically through the analysis of biopsies and cytology samples, in addition to the analysis of colposcopy images. Furthermore, the full benefit of using AI compared to conventional methods alone has not yet been determined.

Therefore, this study aims, through a systematic literature review, to synthesize the best available evidence on the use of generative and integrative AI models as tools to enhance the accuracy of timely cervical cancer diagnostic confirmation in adult women and to compare their effectiveness with traditional diagnostic methods.

## Methods

A scoping review was conducted following the methodology described by Arksey and O’Malley^(11)^ to structure the search and analysis process. First, a clear research question was formulated to define the scope of the study, arising from the need to identify new strategies related to the use of AI models as a tool for the early detection of cervical cancer in adult women. A thorough search was then carried out in academic databases, using key terms and combinations related to the topic.

Predefined inclusion and exclusion criteria were applied to select relevant studies, including both experimental and observational research that provided data on the effectiveness and benefits of this technology. Key information from each study was then extracted and organized. Finally, the results were synthesized in analyses and charts, highlighting the main contributions identified and pointing out areas requiring further research.

Search strategies were developed using Boolean operators, with a focus on the adult female population. The key terms were specifically adapted for PubMed, Scopus, and BIREME databases, ensuring comprehensive coverage of relevant studies. To ensure accuracy in the results, the most appropriate MeSH terms were selected. These terms were then combined using Boolean operators such as AND, to narrow the search, and OR, to broaden the results. Filters were applied based on study type and publication date to refine the search and focus on the most relevant articles. After reviewing the abstracts, the most relevant articles were selected for full-text reading. Finally, the results were organized and filtered to ensure a complete and up-to-date collection of the necessary information for the research. Additionally, the "snowball" method was applied to include references cited in the selected documents that met the inclusion criteria but had not been identified in the initial search. It is important to note that theoretical publications, such as literature reviews, other systematic reviews or meta-analyses, clinical guidelines, letters to the editor, case reports, and documents without access to abstracts or full texts, were excluded.

The selection process for the studies was independently carried out by three researchers across all databases, ensuring consistent results based on the inclusion criteria. The selections were compared to verify their accuracy and agreement.

Subsequently, the included articles were reviewed in full text by all authors, and the following data were extracted into a chart: authors, publication year, study type, type of AI, and summary (Chart 2). A narrative synthesis of the most representative publications included in the review was then conducted.

**Chart 2:** Synthesis of the documents included in the review

Author, year	Title	Study type	Type of AI model	Main findings
Holmström, et al. (2021)^(12)^	Point-of-Care Digital Cytology With Artificial Intelligence for Cervical Cancer Screening in a Resource-Limited Setting	Experimental	Deep Learning	The use of deep learning (DL) for detecting atypia in cervical squamous cells demonstrated higher sensitivity for identifying low-grade lesions and greater specificity for all types of atypia compared to pathologist analysis.
Hou et al. (2022)^(13)^	Artificial Intelligence in Cervical Cancer Screening and Diagnosis	Narrative Review	Machine Learning Deep Learning	Machine learning (ML) and deep learning (DL) reduce diagnostic time and minimize human errors caused by subjective biases, enabling faster and more reliable diagnoses.
Alsalatie et al. (2022)^(14)^	Analysis of Cytology Pap Smear Images Based on Ensemble Deep Learning Approach	Experimental	Ensemble deep learning model, deep convolutional neural network	Ensemble deep learning model, deep convolutional neural network The proposed system analyzes entire slide images and achieves a high level of accuracy compared to existing literature models
Kang and Li (2024)^(15)^	CerviSegNet-DistillPlus: An Efficient Knowledge Distillation Model for Enhancing Early Detection of Cervical Cancer Pathology	Experimental	CerviSegNet-DistillPlus (deep-learning model)	Deep learning offers a more accurate, efficient, and scalable solution than currently available methods, potentially reducing the time between detection and diagnosis.
Lee et al. (2023)^(16)^	Beyond the Microscope: A Technological Overture for Cervical Cancer Detection	Narrative Review	Convolutional Neural Networks (CNNs) Machine Learning (ML) y Deep Learning (DL): Transfer Learning (TL)	CNNs can automate and optimize the interpretation of cervical cytology images, improving the detection of premalignant or malignant abnormalities. They also reduce pathologists’ workload, minimize human errors, and accelerate the diagnostic process.
Wang et al. (2021)^(17)^	Artificial intelligence-assisted fast screening cervical high grade squamous intraepithelial lesion and squamous cell carcinoma diagnosis and treatment planning	Experimental	Cascaded multi-layer deep learning framework	The AI-assisted method outperformed conventional Pap smears and HPV testing, achieving sensitivity comparable to the gold standard provided by pathologists.
Bao et al. (2020)^(18)^	Artificial intelligence-assisted cytology for detection of cervical intraepithelial neoplasia or invasive cancer: A multileft, clinical-based, observational study	Cross-Sectional Observational	Deep Learning	AI-assisted readings showed equivalent sensitivity and higher specificity compared to expert cytologists and superior sensitivity and specificity relative to general cytologists
Benyes et al. (2022)^(19)^	A Comparative Analysis of Deep Learning Models for Automated Cross-Preparation Diagnosis of Multi-Cell Liquid Pap Smear Images	Experimental	Convolutional neural network	Deep learning exhibits high potential for accurately classifying liquid Pap smear images
William et al. (2019)^(20)^	A pap-smear analysis tool (PAT) for detection of cervical cancer from pap-smear images	Experimental	Machine Learning, Fuzzy C-Means	Using ML to classify cervical lesions through cervicovaginal cytology analysis significantly reduced diagnostic time compared to manual analysis, enhancing cervical cancer diagnostic efficiency.
Singh and Goyal (2020)^(21)^	Performance Analysis of Machine Learning Algorithms for Cervical Cancer Detection	Experimental	Machine Learning, Convolutional neural network	The algorithms achieved high accuracy in detecting cervical cancer, with most demonstrating complete sensitivity and specificity, free of false positives or negatives
Alsalatie et al. (2023)^(22)^	A New Weighted Deep Learning Feature Using Particle Swarm and Ant Lion Optimization for Cervical Cancer Diagnosis on Pap Smear Images	Retrospective Observational	Convolutional Neural Networks (CNNs) Ant Lion Optimization (ALO) y Particle Swarm Optimization (PSO) Machine Learning (ML): Support Vector Machines (SVM) y Random Forest (RF)	The combination of deep learning and optimization methods (ALO and PSO) demonstrated high accuracy in classifying seven categories of cervical cancer. This approach could serve as an effective tool for early and precise diagnosis
Hu et al. (2019)^(23)^	An Observational Study of Deep Learning and Automated Evaluation of Cervical Images for Cancer Screening	Case-Control	Deep learning, faster region-based convolutional neural network	Automated visual assessment surpassed traditional methods in accuracy and effectively identified early CIN2 cases
Ito et al. (2022)^(24)^	An artificial intelligence-assisted diagnostic system improves the accuracy of image diagnosis of uterine cervical lesions	Experimental	Convolutional neural network	The AI system achieved higher accuracy in normal cases but lower accuracy for CIN1. Expanding training data slightly improved results, and AI assistance increased gynecologists’ diagnostic accuracy for invasive cancer and CIN2-3.
Stegmüller et al. (2024)^(25)^	Self-supervised learning-based cervical cytology for the triage of HPV-positive women in resource-limited settings and low-data regime	Experimental	MIL (Multiple Instance Learning), SSL (Self-Supervised Learning)	The SSL-based approach demonstrated high precision in identifying abnormalities in cervical cytology, potentially improving triage processes for HPV-positive women and prioritizing patients needing urgent interventions or additional treatments.
Takahashi et al. (2022)^(26)^	Development of a prognostic prediction support system for cervical intraepithelial neoplasia using artificial intelligence-based diagnosis	Experimental	Convolutional neural network	The AI system outperformed conventional colposcopy in terms of accuracy and robustness. AI use can serve as an effective tool to predict CIN2 prognosis based on subtle color tone changes, which are challenging to discern with traditional colposcopy methods
Miyagi et al. (2019)^(27)^	Application of deep learning to the classification of uterine cervical squamous epithelial lesion from colposcopy images combined with HPV types	Retrospective	Convolutional neural network	AI demonstrated potential clinical utility in colposcopy exams, offering benefits to both patients and physicians.
Devi et al. (2023)^(28)^	Prediction and Detection of Cervical Malignancy Using Machine Learning Models	Experimental	Machine Learning (ML): Logistic Regression (LR), Naive Bayes (NB) Deep Learning (DL): Multi-Layer Perceptron (MLP)	ML models showed excellent performance in classifying cervical lesions and accurately predicting cervical cancer.
Mehmood et al. (2021)^(29)^	Machine Learning Assisted Cervical Cancer Detection	Experimental	CervDetect (Machine Learning)	The system accurately predicted cervical cancer and outperformed state-of-the-art studies
Liu and Chu (2024)^(30)^	Analysis of effectiveness in an artificial intelligent film reading system combined with liquid based cytology examination for cervical cancer screening	Cohort Study	Convolutional neural network	The AI system improved cervical lesion detection but required high-quality images and human supervision for complex cases.
Wang et al. (2024)^(31)^	Artificial intelligence enables precision diagnosis of cervical cytology grades and cervical cancer	Randomized Observational Trial	AICCS (combination of a deep-learning neural network and classical machine-learning algorithms)	The system effectively detected abnormal cells and differentiated them from normal cells, potentially leading to earlier detection and intervention
Alsalatie et al. (2023)^(32)^	A New Weighted Deep Learning Feature Using Particle Swarm and Ant Lion Optimization for Cervical Cancer Diagnosis on Pap Smear Images	Experimental	Deep Neural Networks (DNN) Ant Lion Optimization (ALO) y Particle Swarm Optimization (PSO)	The combination of PSO and ALO optimization methods with deep learning significantly enhanced accuracy and efficiency in diagnosing cervical cancer using Pap smear images. This approach outperformed traditional methods, demonstrating its potential as a superior tool for early and precise cervical cancer diagnosis

## Results

**Figure 1:**
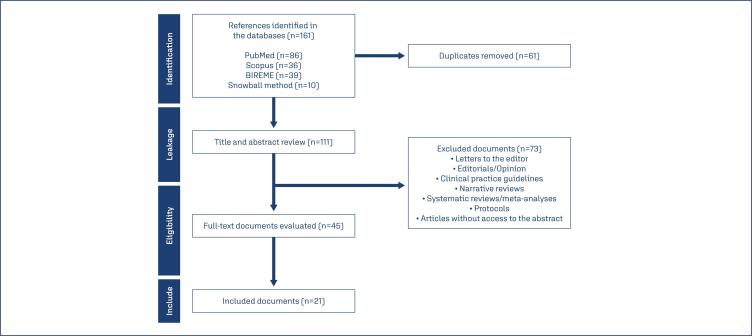
Flowchart of studies

The initial analysis was conducted using the Rayyan system for study selection, resulting in the inclusion of 161 articles. After removing duplicates, 111 articles were retained. A filter was then applied using the keyword "diagnosis," which yielded 45 articles for full-text review. These were evaluated based on their methodology, results, and relevance to the research question. Taking this into account, 24 articles were excluded, ensuring that only relevant studies were included. Ultimately, 21 suitable articles remained, along with 10 additional articles identified through snowball sampling. All files were obtained and fully reviewed by the research team. The documents were then categorized into three groups: [1] applicability of AI in cervicovaginal cytology, [2] applicability of AI in colposcopy, and [3] applicability of AI in biopsy. The review followed the PRISMA-ScR guidelines for scoping reviews (Figure 1).

Cervicovaginal cytology is the primary screening method for cervical cancer worldwide; however, it has significant limitations, which are discussed in section number 4. It has been shown that these limitations can delay early diagnosis and the timely initiation of treatment. Given this, various studies have been conducted to determine the utility of AI in improving this process.

Through our literature review, we found that the most commonly used AI method by researchers is Deep Learning (DL), which was selected in 9 of the articles analyzed. These studies demonstrated that its use in conjunction with cervicovaginal cytology reduced the workload of pathologists and shortened the time required for analysis.^(12–15)^ Additionally, it showed sensitivity and specificity for detecting cervical cell atypia that was similar to or even greater than the analysis performed by pathologists alone,^(12,13,15,17,18)^ as well as high accuracy for cervical cancer diagnosis.^(13–15,19)^ Following this, there is the study of the applicability of ML in conjunction with conventional cervicovaginal cytology, which was the subject of investigation in 6 of the articles reviewed. It was found that ML reduced the time required for cervical cancer diagnosis and demonstrated good accuracy in detecting premalignant and malignant lesions.^(13,15,20,21)^ Other types of AI studied both individually and in combination, such as CNN with high accuracy for cervical cancer diagnosis,^(14,15,19,21–24)^ and other methods like Self-Supervised Learning (SSL) and Multiple Instance Learning (MIL), which showed high accuracy in identifying anomalies in cervical cytology.^(25)^

Colposcopy is an imaging technique used for diagnosing and classifying cervical lesions. When a positive result is obtained from a screening test, a biopsy is necessary, which means that the gynecologist, through colposcopy, must perform a detailed visual examination of the cervix to identify the optimal site for sample collection, ensuring an appropriate histopathological assessment and determining whether further treatment is required. However, this diagnostic tool has low reproducibility, as its accuracy largely depends on the specialist's experience. Additionally, the training period is long, and there is a shortage of trained personnel. For this reason, less experienced colposcopists often struggle to identify the area with the highest concentration of lesions, leading to misclassifications in up to half of cases. Inaccurate techniques can also cause potential harm, such as pain, infection, bleeding, and others.^(13,26)^

Thanks to advancements in AI, multiple deep learning-based systems have been developed, which use ML algorithms for image processing. These systems allow the training of specialized models, optimizing the accuracy and efficiency of cervical cancer diagnosis.

As Takahashi et al.,^(26)^ trained an AI algorithm using a dataset of 593 images from patients diagnosed with grade 2 cervical intraepithelial neoplasia (CIN2), they developed a convolutional neural network capable of automatically detecting high-grade cervical lesions in the uterovaginal area. The developed system achieved an overall correct response rate of 89.7%, with the number of high-grade lesions detected being comparable to the number detected by experienced colposcopist gynecologists. Additionally, based on the obtained results, the authors suggest that through the analysis of imaging findings, their AI system could contribute to the prognosis of pathological lesions, as it was found that the percentage of high- grade lesions present was significantly related to the progression of CIN2 lesions. This could enable timely treatment and prevent progression to invasive cancer.^(26)^

Similarly, Miyagi et al.,^(27)^ developed a classifier system using a convolutional neural network to predict pathological diagnoses based on images of cervical squamous intraepithelial lesions (SIL) combined with HPV types. The system achieved an accuracy of 0.941 on the test set, while gynecological oncologists achieved an accuracy of 0.843. Although the two methods could not be directly compared due to differences in the lesions identified, the results showed that the system was not inferior to conventional colposcopy, as both methods produced similar results when diagnosing high-grade SIL (HSIL) and low-grade SIL (LSIL). However, since the classifier system was not trained with images of cervicitis, adenocarcinoma, and invasive cancer, these cases were diagnosed only by colposcopists and were ignored by the AI. This highlights a limitation in the detection and classification capabilities of intelligent systems, as their performance is closely linked to the images used during the training process.^(27)^

Biopsy in cervical cancer screening is a diagnostic procedure used to confirm the presence of precancerous lesions or invasive cancer in the cervix. This procedure is generally performed after abnormalities are detected in initial screening tests, such as the Papanicolaou (Pap) test or HPV detection tests. Recently, advancements in AI, specifically in deep learning algorithms, have revolutionized the way cervical cancer is analyzed.^(16)^ In a study conducted by Hu et al.,^(23)^ a deep learning algorithm called Faster R-CNN, trained with digitized cervigrams, was used to perform an automated visual assessment (AVA) of the cervix. This algorithm demonstrated that AVA outperforms traditional methods in accuracy and is capable of effectively identifying early cases of CIN2, detecting 55.7% of precancers in women aged 25 to 49 with a single round of screening.^(23)^

Based on the reviewed literature, an objective analysis was conducted of the results obtained in the different studies to compare the AI methods used by researchers. To do this, the percentage data for sensitivity, specificity, and accuracy from the articles that provided at least one of these metrics were synthesized and then averaged to obtain a unified value (Chart 3). This revealed that the AI method used individually with the best results was ML, with an average sensitivity of 0.954 and specificity of 0.985. The Convolutional Neural Network (CNN) demonstrated the highest accuracy, with an average value of 0.990. However, the use of Deep Learning combined with CNN achieved the highest sensitivity, with average values of 0.991 and 0.992, respectively, and the highest accuracy, with average values of 0.995 and 0.994, respectively.

**Chart 3:** Comparison of Sensitivity, Specificity, and Accuracy of Different Types of AI

Type of AI	Reference	Sensitivity	Average sensitivity	Specificity	Average specificity	Accuracy	Average accuracy
DL	14	0.942	0.949	0.894	0.957	0.927	0.893
	16	0.899		1.000		0.929	
	11	0.957		0.847		-	
	15	0.928		0.910		0.823	
	17	1.010		1.260		-	
	26	0.956		0.833		-	
	30	0.991	0.991	0.996	0.996	0.995	0.995
ML	27	0.873	0.954	-	0.985	0.863	0.943
	15	0.998		0.996		0.984	
	19	0.993		0.975		0.989	
	28	-		-		0.936	
CNN	18	-	0.8377	-	0.758	0.990	0.990
	22	1.000		0.575		-	
	29	0.675		0.941		-	
DL + CNN	28	1.000	0.992	-	-	0.996	0.994
	31	0.983		-		0.991	

DL -Deep Learning; ML - Machine Learning; CNN - Convolutional Neural Networks

## Discussion

AI is increasingly being integrated into medicine, particularly in diagnostic processes, providing more stable, accurate, and efficient tools. This integration has led to improved outcomes compared to conventional methods, significantly reducing the time required for detection and management implementation.^(15)^ The gold standard for cervical cancer diagnosis is the molecular detection of human papillomavirus (HPV) in cervical cells. However, current diagnostic methods still often rely on visual analysis, which is heavily dependent on the expertise of the pathologist or oncologist and is subject to human error, thereby reducing diagnostic efficiency.^(14)^ AI has demonstrated excellent applicability in cervical cancer diagnosis. AI possesses the ability to automatically recognize images and patterns, extract features, and process data using advanced technological algorithms.^(13)^

Cervicovaginal cytology is the most widely used cervical cancer screening method globally, allowing for the detection of cancerous and precancerous lesions that can be treated in time to prevent the development of malignant conditions.^(16)^ This screening method has reduced cervical cancer mortality by 70%, yet it presents significant limitations in its application. It requires substantial time investment and trained personnel to manually review each slide individually and in detail, making the results subjective and subject to interobserver variability. Additionally, it is highly prone to human error, with low sensitivity and specificity.^(12,16)^ Therefore, given the substantial technological advancements since the introduction of cervicovaginal cytology in the 1970s, optimizing the early detection of premalignant and malignant lesions has become crucial to improving the prognosis of affected patients. From this perspective, the need arises to improve outcomes and accelerate the process of conventional cervicovaginal cytology. Consequently, various approaches have been proposed to leverage AI to achieve this goal. An extensive review of the current literature suggests that integrating AI models with cervicovaginal cytology as a conventional screening and diagnostic method can significantly enhance the timely diagnosis and treatment of patients with cervical cancer. This improvement is attributed to AI's ability to reduce the time required for diagnosis through the digitization of samples, enabling immediate analysis and facilitating the prompt initiation of treatment, thereby improving patient prognosis. Furthermore, AI alleviates the workload of pathologists and minimizes interoperator subjectivity in results, ultimately contributing to greater diagnostic accuracy.

Regarding colposcopy, the integration of AI into this diagnostic procedure has demonstrated greater accuracy and reliability compared to conventional colposcopy, achieving results that are even comparable to those of gynecologic oncologists. As a result, multiple AI systems currently under development have the potential to be employed by both experienced gynecologists and those in training for colposcopy. This integration can significantly enhance the precision of diagnosis and the classification of cervical lesions.^(26,27)^ It would also reduce the risk of misdiagnosis, facilitate earlier management, and mitigate the procedural risks involved.^(13)^

Regarding biopsy, although significant advancements have been made in the use of AI for cervical cancer diagnosis through other detection techniques, the application of AI in this invasive procedure remains limited. The literature reviewed revealed a scarcity of studies exploring the direct implementation of AI in biopsy interpretation. However, one previously mentioned article integrates a Faster R-CNN system, which demonstrated high sensitivity in detecting precancerous lesions (CIN2+). This approach outperformed colposcopies in analyzing the same images, enhancing diagnostic accuracy, and showing favorable comparisons with traditional Pap smear testing and PCR-based HPV testing. Although automated visual evaluation (AVE) holds great potential, its practical implementation faces several challenges.^(23)^ For instance, digital imaging technology and modern cameras are not yet widely adopted for capturing high-quality images at the point of care. Additionally, false positives remain a significant issue, as the algorithm may identify visual anomalies that do not necessarily indicate cancer.

Finally, it is crucial to emphasize the impact of AI implementation in the early diagnosis of cervical cancer on healthcare systems. Employing these tools and technological strategies could help bridge gaps in access to advanced diagnostics, particularly in rural areas or regions with limited access to healthcare services. This would enable broader screening and diagnostic coverage in areas lacking specialized professionals, equipment, and resources necessary for sample collection and processing.^(12,16)^ However, this review has certain limitations. There is still a limited number of studies exploring the use of AI in colposcopy and cervical biopsy, especially when compared to its application in cytology. Further research is needed to evaluate its integration with other imaging and biochemical diagnostic tools, as well as its potential to model risk prediction and prognosis. Despite a comprehensive review, available evidence remains scarce, particularly when comparing the diagnostic performance of AI versus human interpretation.

## Conclusion

In this exploratory review, articles on the integration of AI into traditional cervical cancer diagnostic methods were analyzed. It was found that these tools, especially ML, can improve the diagnostic accuracy of cytology, colposcopy, and biopsy. However, further studies are needed to continue evaluating the applicability of AI, particularly in association with colposcopy and cervical biopsy, as research in these areas remains limited. Additionally, more investigation is required to enhance the accuracy of these systems. For instance, in the development of algorithms that rely on image-based training, it is essential to include a variety of cervical cancer stages, avoiding a focus on just one stage throughout the study process. Other important areas for future research include the integration of AI with complementary imaging techniques, such as tomography and magnetic resonance imaging, for monitoring and assessing cervical cancer, as well as its application in molecular testing and the identification of specific biomarkers for this pathology. Thanks to its exponential growth, AI is expected to become increasingly integrated into the medical field, particularly in diagnostic methods, such as those used for cervical cancer. This is due to its ability to reduce detection time with high precision and even identify precancerous lesions, enabling the implementation of timely treatment by prioritizing high-risk patients and thereby reducing the morbidity and mortality burden in this population. Moreover, AI could lower the costs associated with managing this condition by decreasing the workload for specialists and reducing the need for highly trained personnel. Early detection would also contribute to lowering the costs of treating advanced-stage disease. AI offers a significant opportunity to expand and equalize access to early detection in remote areas lacking adequate medical infrastructure or specialist availability. This would allow more women, regardless of their location, to access screening and early diagnostic services at a lower cost and without needing to travel to urban centers. Based on the current literature, AI appears to offer potential benefits, particularly in the early detection of cervical cancer; nevertheless, additional research is needed to support its integration into diagnostic protocols.
